# Policy brief: adaptive cycling equipment for individuals with neurodevelopmental disabilities as durable medical equipment

**DOI:** 10.3389/fresc.2023.1160948

**Published:** 2023-06-05

**Authors:** Mary E. Gannotti, Margaret E. O’Neil, Maria Fragala-Pinkham, George E. Gorton, Daniel G. Whitney

**Affiliations:** ^1^ Department of Rehabilitation Sciences, University of Hartford, West Hartford, CT, United States; ^2^ Department of Physical Therapy & Kinesiology, University of Massachusetts Lowell, Lowell, MA, United States; ^3^ Department of Physical and Occupational Therapy, Boston Children’s Hospital, Boston, MA, United States; ^4^ Department of Research, Shriners Hospitals for Children, Springfield, MA, United States; ^5^ Department of Physical Medicine and Rehabilitation, University of Michigan, Ann Arbor, MI, United States

**Keywords:** neurodevelopmental disability, adaptive cycling, adaptive tricycles, policy, payment, cerebral palsy, autism, Down syndrome

## Abstract

- Durable medical equipment (DME) policies require that the equipment be medically necessary; however, adaptive cycling equipment (bicycles and tricycles) are usually not deemed medically necessary.

- Individuals with neurodevelopmental disabilities (NDD) are at high risk for secondary conditions, both physical and mental, that can be mitigated by increasing physical activity.

- Significant financial costs are associated with the management of secondary conditions.

- Adaptive cycling can provide improved physical health of individuals with NDD potentially reducing costs of comorbidities.

- Expanding DME policies to include adaptive cycling equipment for qualifying individuals with NDD can increase access to equipment.

- Regulations to ensure eligibility, proper fitting, prescription, and training can optimize health and wellbeing.

- Programs for recycling or repurposing of equipment are warranted to optimize resources.

## 1. Introduction

Approximately 17% of the population in the United States (US) is comprised of individuals with neurodevelopmental disabilities (NDD), such as cerebral palsy (CP), intellectual disability (ID), autism (ASD), and Down syndrome (DS) (1), and the prevalence of NDD is on the rise with a 10% increase from 2009 to 2017 (1). Lifetime direct and indirect health care costs for individuals with NDD are significant, with estimated expenses at $11.5 billion (in 2003 dollars) for persons born in the US in 2000 with a diagnosis of CP (2). Health care utilization (e.g., emergency room and outpatient visits, psychiatric hospitalizations, and rehabilitation care) is double for individuals with NDD compared to peers without NDD and even higher for those with epilepsy and poor physical and mental health (3–8). Health care costs for this population are ***potentially modifiable by***: 1) preventing or delaying the onset of secondary comorbidities and 2) reducing the risk of early-onset chronic diseases ***through increasing physical activity (PA)***.

Addressing the burden to society, social and financial, of the management of secondary conditions of adults with NDD is a ***public health crisis that warrants immediate attention and action***. A growing body of literature highlights the increased prevalence of mental and physical conditions (5, 9–13), as well as polypharmacy (14), among adults with NDD. Research has demonstrated that the cost of care for adults with NDD is significantly greater than other groups. Estimates for the care of adults with intellectual or developmental disability (IDD) are 36% higher than peers in Canada and 20% higher in the US (3, 15). The lifetime cost of care for an individual with CP is estimated to be 80% higher than peers without CP in South Korea (16). Economic evaluations support overall cost effectiveness of interventions to support individuals with NDD—targeted interventions reduce costs to the system and increase quality of life (17). Given that regular PA can directly address mental health and physical health, ***policy that directly supports PA for individuals with NDD is warranted***.

A clear dose-response relationship exists between participation in PA and chronic diseases (18, 19). Increasing participation in PA (20, 21) with a target of 150 min per week (22) decreases the prevalence of chronic disease. Physical activity has demonstrated positive effects on the health and wellbeing of individuals with NDD (23–25).

Physical activity is most feasible for people with functional disability when broken up into 25–30-minute bouts throughout the week (20). Walking is the most common PA reported by adults with mobility disorders (21); yet many environmental barriers are reported (26). Individuals with NDD often encountered lack of accessible equipment, lack of expertise in adapting activities, and concerns for safety when utilizing programs and spaces open to the general public (27–29). Hence, many individuals with NDD lack the supports for lifelong PA.

Physical activity can be improved for individuals with NDD in a variety of ways; adapted programs for swimming, cycling, dancing, walking, and sports clubs exist throughout the US (see: https://www.nchpad.org/). Adaptive cycling equipment (AdCE), which includes a variety of specialized bicycles and tricycles, can provide access to a form of PA for people who are unable to walk or who cannot ride a two-wheeled bicycle due to cognitive or physical impairments. Adaptive cycling can be performed in community spaces, such as rail trails, sidewalks, and bike lanes, providing a means of PA that is integrated in daily routines and inclusive. (30).

Currently, access to AdCE is limited due to cost and not being classified as durable medical equipment (DME) by the Centers for Medicaid and Medicare Services (CMS) or other insurance carriers. Adaptive cycling equipment is available to those who can afford the cost, have procured money from charitable organizations, or have had success with state administered programs. Advocating for AdCE as DME for individuals with NDD is both an ethical and a pragmatic issue. Steps must be taken to modify costs of chronic disease among individuals with NDD and access to opportunities for PA must be equitable.

The combination of increased prevalence of NDD (1) with increased survival into middle and late adulthood (31) forecasts an even greater financial burden for health care costs than previously experienced. Given that a portion of the cost is modifiable by PA, policy to support PA for this population is warranted. Additionally, given that environmental barriers contribute to decreased opportunities for PA, providing mechanisms for supportive equipment is a health equity issue. The purpose of this brief is to provide a rationale for expanding health policy to include AdCE as medically necessary for individuals with NDD and discuss potential impacts of such policy changes.

### 1.1. Adaptive cycling equipment and neuro-developmental disabilities

Adaptive cycling is an alternative form of PA for people for whom 150 min of PA a week (20) via locomotion is not feasible in a wheelchair, gait trainer or other assistive device. Adaptive cycling equipment can be individualized by physical therapists and other rehabilitation professionals with expertise in PA and NDD to provide safe and functional PA (32). For people with decreased physical or cognitive abilities, adaptive cycling is generally easy to learn and there is a low risk of falls. The participation benefits afforded by adaptive cycling are important for maintaining motivation for ongoing use of AdCE (33). Having a PA plan that includes adaptive cycling that is developed or supervised by a rehabilitation professional is another way to achieve goals for a safe and effective PA dose (frequency, intensity and time) using the AdCE. It is our position that the ***potential fun*** (34) to be had with AdCE should promote adherence and use, and ***facilitate the goal*** of increasing PA to mitigate chronic disease.

Table 1 highlights systematic reviews that summarize the positive impact of adaptive cycling on individuals with NDD. Individuals with ID or ASD who participate in adaptive cycling demonstrate increased PA, reduced body fat (35) and improved physiological outcomes (36), cognition (37), and gross and fine motor skills (37, 38). Participation in adaptive cycling reduces depression and improves social coping skills among individuals with NDD (39–42). Adaptive cycling can provide increased means of mobility, such as a means of transportation to and from work and for activities of daily living (43). Cycling can improve engagement in the community and quality of life (33). It is important to note an appropriate dose of activity (frequency, intensity, and time) is critical in achieving desired outcomes (44).

**Table 1:** Systematic reviews of adaptive cycling and neuro developmental disabilities.

Author, year	Population	Research question	Number of studies identified	Findings
Armstrong et al, 2019 (45)	Cerebral palsy	Efficacy of cycling to improve function and reduce activity limitations in children with cerebral palsy; the optimal training parameters for improved function; and whether improvements in function can be retained	9 studies containing data on 282 participants met full inclusion criteria	Cycling can improve muscle strength, balance and gross motor function in children with cerebral palsy; however, optimal training doses are yet to be determined. There was insufficient data to determine whether functional improvements can be retained
Catelli et al, 2019 (42)	Cerebral palsy	Effects of the cycle ergometer on the gross motor function of children with cerebral palsy by the Gross Motor Function Measure	3 articles met inclusion criteria. Children with CP (*n* = 127) classified at Gross Motor Function Classification Sysstem Levels I-V and ages 6 to 18 years were included in the studies.	Improvements in gross motor function were equivalent for cycling and physical therapy interventions.
Fragala-Pinkham et al, 2021 (44)	Intellectual disability (Down syndrome and Autism Spectrum Disorder)	Efficacy of lower extremity cycling interventions for youth with intellectual disability (ID)	8 articles met inclusion criteria. Children and young adults, 7–26 years (*n* = 229), with diagnoses of Down syndrome, autism spectrum disorder, or unspecified ID participated in the studies.	Moderate to weak evidence exists to support two wheeled cycling instructional programs or stationary cycling interventions for children and young adults with intellectual disabilities.
Thevarajah et al, 2022 (30)	Developmental disabilities	Effects of adapted bicycle riding on body structures and functions, activity, participation, and quality of life outcomes in children with disabilities, along with family-level participation outcomes	10 articles met inclusion criteria. Participants (*n* = 234) were between 4 and 18 years of age with diagnoses of CP (*n* = 66), Down syndrome (*n* = 102), autism spectrum (*n* = 59) and intellectual disability (*n* = 7).	Some evidence that adapted bicycle riding interventions improve gross motor function and leg muscle strength and promote PA.

Among children with CP, there is a body of evidence to support that cycling has physical benefits such as improved endurance, strength, gross motor function, gait function, and bone health (30, 38, 42, 45–54). Improvements in gross motor function have been reported in children with CP who are non-ambulatory (47, 50) and ambulatory with bilateral or unilateral involvement (51, 53, 54). Changes in bone mineral density at the distal femur (49) hold promise for mitigating nontraumatic fractures (55, 56). Similar to individuals with DS, ASD and/or ID, dose of activity (frequency, intensity, and time) was noted to be important in achieving desired outcomes for individuals with CP (45).

## 2. Policy options and implications

### 2.1. Equipment to support physical activity is medically necessary

The rationale to support AdCE as medically necessary is that regular PA and socialization can mitigate secondary conditions associated with increased sedentary behavior (20), which in turn, can reduce the cost to the individual, family and society. Because individuals with NDD are at risk for more and earlier onset of limitations in PA and incur a greater cost of health care than their peers without NDD, specialized equipment is warranted to provide opportunities for PA. Considering AdCE as medically necessary for individuals with NDD also addresses an issue of equity, as the lack of social supports and the built environment further restrict opportunities for PA.

### 2.2. Adaptive tricycles and bicycles are durable medical equipment

The Centers for Medicare & Medicaid Services (CMS) provide the regulatory guidance for reimbursement of DME. For a device/piece of equipment to be considered DME, it must be: (1) durable, (2) used for a medical reason, (3) not generally useful for someone who is not sick or injured, (4) used in the home, and (5) expected to have a lifetime of 3–5 years (57).

Adaptive cycling equipment, we argue, meet these criteria. They are used for a medical reason among individuals with NDD, as the equipment is used to reduce the risk for chronic disease and early-onset chronic disease by increasing opportunities for PA. We argue that there is sufficient evidence to support the positive physical benefits of individuals with NDD (Table 1). In addition, we argue that adaptive cycles are generally not useful to individuals without cognitive, physical, or behavioral impairments, and NDD as a diagnostic group have great health disparity in outcomes and PA as compared to peers without NDD.

Adaptive cycling equipment is not listed on the DME Reference list (57). There is no specific Healthcare Common Procedure Coding System (HCPCS) code for AdCE, and A9300- exercise equipment is generally not covered as part of any of the Medicare or Medicaid programs (58). In the Medicare Claims Processing Manual, Chapter 20, “Durable Medical Equipment, Prosthetics and Orthotics, and Supplies” (57), continuous passive movement machines are the only exercise equipment listed as a benefit. Durable Medical Equipment HCPCS Codes ranging from E0100-E8002 include a range of locomotion supports (58), none of which include AdCE. In rare cases, payment from insurance companies may occur and the HCPCS Code E1399 DME Miscellaneous can be used.

Medicaid's Home & Community-Based Services 1915(c) & 1915(i) allow states to choose groups of people with particular needs and health conditions to receive tailor-made healthcare options at home or within the community (59). In each state, the Department of Health and Human Services and Department of Developmental Disabilities administrate programs that allow for special programs for individuals with NDD. Policy implementation varies from state to state. Some states, like Rhode Island, cover therapy related equipment, but explicitly state that AdCE is “not primarily medical in nature” and not covered (60). The need for AdCE is evaluated on a case-by-case basis in other states and frequently denied. Reasons for denial include that AdCE is recreational and not medical, other forms of equipment for exercise are less costly, that the AdCE can be used in school or physical therapy sessions, and that the cycling equipment cannot be used in inclement weather.

**Figure 1:**
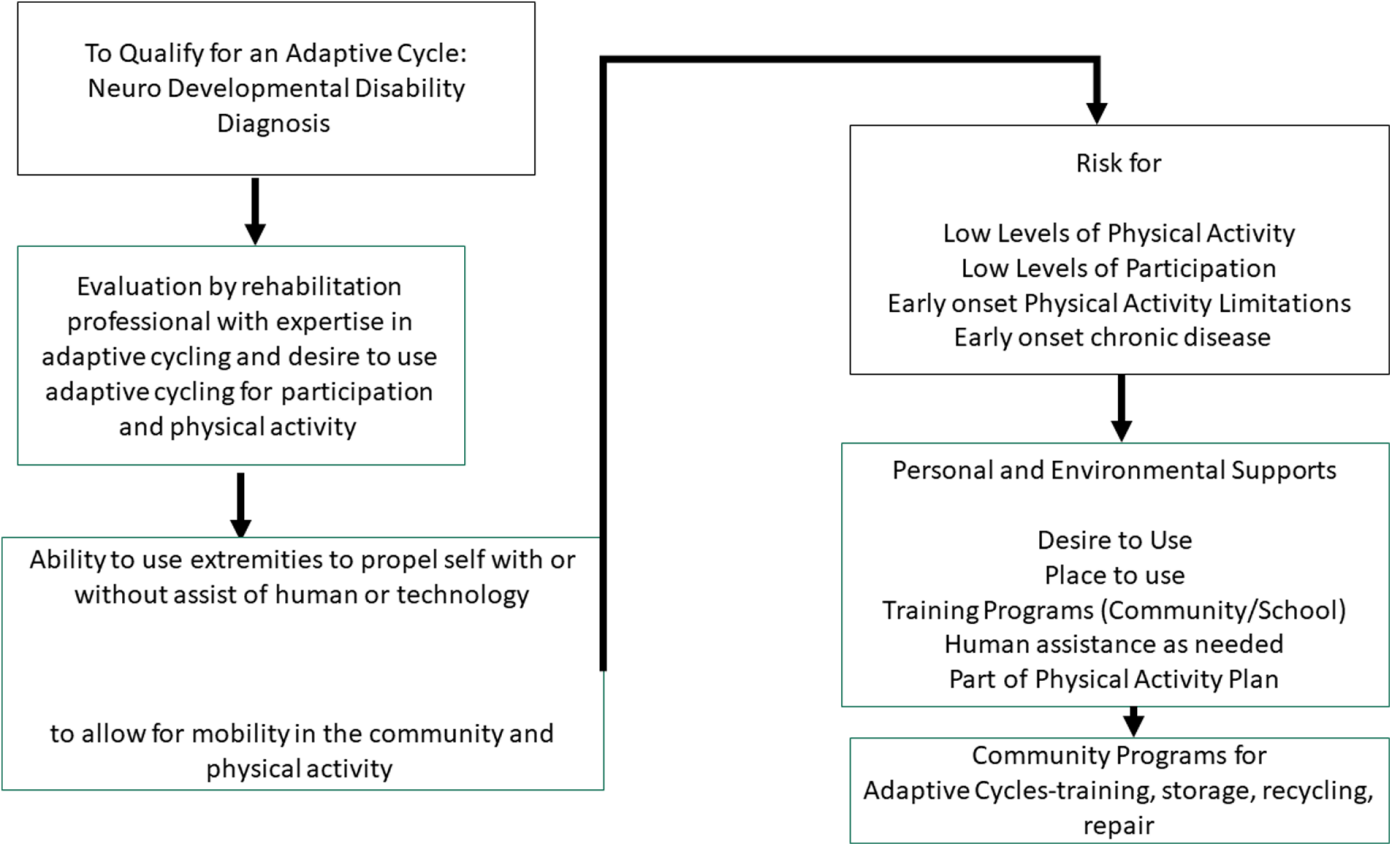
Adaptive cycle eligibility criteria.

It is our position that the benefits of AdCE outweigh the costs, and AdCE is medically necessary and of benefit. We argue that CMS should expand the definition of DME to include AdCE to support PA among qualifying individuals with NDD. The following criteria are proposed for qualifying for service (see Figure 1):

1) Have a diagnosis of a Neuro Developmental Disability

The target population is individuals with NDD, given the high cost of care and the exponentially growing population with high cost associated with secondary conditions. Focusing policy on this segment of the population is proactive and will provide the most benefit as there is the greatest need.

 a. The presence of a functional mobility limitations that will limit capacity for PA is required for eligibility

This aligns with recommendations for PA for people with disabilities and national initiatives to increase PA (20). The individual should require an additional form of PA besides walking to obtain the recommended dose of PA. This acknowledges that walking is not accessible to all individuals with NDD, and adaptive cycling may suit this need.

2) Evaluation by a rehabilitation professional with expertise in adaptive cycling and designing exercise programs to break up sedentary behavior (total and incremental bouts).

Expertise in AdCE is necessary to choose, adapt, modify, and prescribe the most appropriate device. The professional will address the necessary safety features given the individual's needs/abilities, and the environment in which the cycle will be used. Adaptive cycling should be part of an overall plan to increase PA designed in collaboration with a rehabilitation professional. Use of a helmet and other safety mechanisms will be part of the evaluation and prescription.

3) Demonstrate ability to use extremities to propel self with or without assist of human or technologic assistance and
   a. Evidence of muscle activation throughout limbs and trunk when cycle moves;
   b. Increased heart rate when cycling as compared to quiet sitting

If an individual can produce muscle activation to move the adaptive cycle, either in the presence or absence of assistance, and can produce an increase in heart rate, physiological processes will be activated to improve health and well-being.

4) At high risk for chronic disease and sedentary lifestyle

Given the individual's overall health status, risk for chronic disease or early-onset chronic disease must be weighed in as a greater cost than AdCE. The cost of AdCE and training for adherence to a cycling program should be a lower health cost than management of chronic conditions.

5) Personal and environmental supports

Individuals with NDD who qualify for health benefits to obtain AdCE would need to demonstrate a place to use and store the AdCE (either in home or community), the availability of human assistance as needed, and the availability of community, hospital or school programs for training. Additionally, proper use of safety equipment, such as a helmet, would be required. Increased participation in the community facilitates motivation for use and increases likelihood of achieving therapeutic dose of activity. Community supports may be required for training, storage, use, and repair (see Figure 1). An upstream outcome of this criteria is that heightened awareness and increased participation of individuals with NDD in community programs, which will drive programs to be more inclusive and adaptive (use universal design).

### 2.3. Actionable recommendations

The actionable recommendations from this brief include three possibilities as summarized in Table 2: (1) regulatory changes occurring at CMS concerning the AdCE as DME for individuals with NDD, (2) legislative action to enact changes in CMS policy about AdCE and individuals with NDD; and (3) no regulatory or legislative changes, with continued advocacy and research.

**Table 2:** Summary of alternative actions.

Action	Summary
Regulatory changes	Change would occur in the health care insurance policies directly- Centers for Medicare & Medicaid Services
Legislative	Amendment to CHRONIC Care Act passed through Congress would instigate change in the Centers for Medicare & Medicaid Service policies
No Change	Centers for Medicare & Medicaid Service policies will remain, legislation would not be passed; research and advocacy efforts would continue.

#### 2.3.1. Regulatory

A regulatory change would require CMS to change current policies about DME to include coverage of AdCE as a covered benefit for individuals with NDD. National coverage determinations (NCD) are made using and evidence-based review process to support medical necessity and include input of the public (61). In the absence of an NCD, states can make a local coverage determination (LCD). The process of requesting an NCD involves a written request that is submitted to CMS (61) that provides a clearly defined beneficiary group, a clear purpose and rationale, and specific item to be covered. Requests for NCD undergo a structured review process, which can take about 9 months (61). Any regulatory changes would require development of appropriate HCPCS codes for AdCE.

#### 2.3.2. Legislative

The “Creating High-Quality Results and Outcomes Necessary to Improve Chronic (CHRONIC) Care Act” signed in 2018 was designed to advance organized person-centered care for people with complex care needs (62). The law permanently authorizes Special Needs Plans to target and serve high need and high-risk beneficiaries (63). The CHRONIC Care Act expands and adapts the supplemental benefits to meet the needs of Medicare Advantage enrollees and promotes integrated care among dual eligible Special Needs Plans enrollees (63) Legislative amendments to the CHRONIC Care Act could support adaptive equipment to promote PA for individuals with NDD as medically necessary. These amendments would require CMS to undergo regulatory changes as described above to provide coverage for AdCE, but these changes would be supported by legislation. Policies concerning other types of adaptive equipment and associated costs of health care professional supervision used to promote PA among individuals with NDD would also require review and policy change.

#### 2.3.3. No action

No action would involve neither changes to CMS regulatory policies concerning AdCE for individuals with NDD nor amendments to CHRONIC Care Act. Several systematic reviews support the physical and mental health benefits of AdCE for use by individuals with NDD (30, 42, 44, 45); and future investigations will provide more evidence for increasing participation, wellbeing, as well guidelines for recommended use and dosage for optimal benefits. Advocacy efforts will continue on a case-by-case basis to use the current evidence to justify AdCE to third party payers. Health care costs for individuals with NDD will continue to rise in the US, as programs and policies do not support preventative care.

### 2.4. Implications

The strength of this proposed policy change is that it provides a strategy towards meeting the national goal of increasing PA of individuals with NDD (20, 64). Lack of action is not an option given the increasing longevity of individuals with NDD (1), the increasing numbers (1), and the high prevalence and cost of non-communicable diseases (65–67). There is a need for targeted policy changes, and this proposal provides a way to increase access to needed equipment. If this policy proposal is adopted, AdCE will become more accessible to individuals with NDD. This will in turn, increase consumer demand for space and programming, producing pressure to change other sectors of the environment which limit access to PA.

A weakness of this proposed policy is that there is little assurance that individuals with NDD who need training will be provided that training when the AdCE is obtained. Uniform policy to support programs for adaptive cycling and adaptive cycling programs do not exist, and given geographic location access to programs will vary. Support of a physical therapist or another rehabilitation professional with expertise in adaptive cycling is needed to ensure individuals who receive AdCE have the proper training and programming. Another weakness is that not all physical therapists and rehabilitation professionals have expertise in adaptive cycling, and currently no certification exists for professionals around adaptive cycling. This may make it difficult for consumers to identify qualified providers. Additionally, providing expert recommendations for adaptive cycling is currently not billable time for clinicians. Time for assessment and recommendations is needed for the adaptive cycle to be personalized for optimal function. This would require additional policy changes.

There are many opportunities for the policy proposal to have a positive impact. Existing organizations and programs that support adaptive cycling and sports for individuals with NDD that are community-based and spread throughout the nation could expand their programming and training. Adaptive cycle exchange and fitting programs could be developed in collaboration with these programs. There are many opportunities for inclusive community activities using community bike paths. Creating opportunities using community bike paths or sidewalks for individuals with NDD to cycle to work or to the store could be included in community initiatives for healthy living and inclusion.

There are many real threats for the policy proposal not to have the desired impact. If an individual does not regularly perform cycling as part of an overall PA plan, the exercise may not mitigate a substantial portion of the risk for chronic disease. Reasons for lack of time spent exercising can be related to illness, motivation, and inability to use the AdCE. Individuals who are in apartments or multilevel homes may not be able to store the equipment on their property, and having the equipment at school or the clinic limits access. Some individuals need to travel to bike paths to use the equipment, and may have difficulty transporting the AdCE. Additionally, AdCE needs maintenance or repairs which may be difficult to complete without support. As individuals change, there may be the need for a different type of AdCE. Programs that provide support for maintenance, storage, and exchange of cycles could address these threats.

## 3. Conclusion

Policy change to include AdCE as DME for individuals with NDD is one step to address the growing cost of care of children and adults with NDD (3, 15). It is an ethical dilemma, as individuals with NDD are at a greater risk than peers for non-communicable diseases (65, 68) and have more barriers to performing PA (27). Strategies identified by the Centers for Disease Control to increase PA of people with disabilities include a broad array of community recommendations, along with individual supports, built environment, community programming, and community prompts (64). Increasing access of individuals with NDD to AdCE could have positive benefits on increasing PA (44, 45) and may mitigate lifetime costs of care and improve quality of life.
